# Transglutaminase 3 negatively regulates immune responses on the heart of the mosquito, *Anopheles gambiae*

**DOI:** 10.1038/s41598-022-10766-z

**Published:** 2022-04-25

**Authors:** Yan Yan, Abinaya Ramakrishnan, Tania Y. Estévez-Lao, Julián F. Hillyer

**Affiliations:** Department of Biological Sciences, Vanderbilt University, VU Station B 35-1634, Nashville, TN 37235 USA

**Keywords:** Entomology, Innate immunity, Cardiovascular biology

## Abstract

The immune and circulatory systems of insects are functionally integrated. Following infection, immune cells called hemocytes aggregate around the ostia (valves) of the heart. An earlier RNA sequencing project in the African malaria mosquito, *Anopheles gambiae*, revealed that the heart-associated hemocytes, called periostial hemocytes, express transglutaminases more highly than hemocytes elsewhere in the body. Here, we further queried the expression of these transglutaminase genes and examined whether they play a role in heart-associated immune responses. We found that, in the whole body, injury upregulates the expression of *TGase2*, whereas infection upregulates *TGase1*, *TGase2* and *TGase3*. RNAi-based knockdown of *TGase1* and *TGase2* did not alter periostial hemocyte aggregation, but knockdown of *TGase3* increased the number of periostial hemocytes during the early stages of infection and the sequestration of melanin by periostial hemocytes during the later stages of infection. In uninfected mosquitoes, knockdown of *TGase3* also slightly reduced the number of sessile hemocytes outside of the periostial regions. Taken altogether, these data show that *TGase3* negatively regulates periostial hemocyte aggregation, and we hypothesize that this occurs by negatively regulating the immune deficiency pathway and by altering hemocyte adhesion. In conclusion, *TGase3* is involved in the functional integration between the immune and circulatory systems of mosquitoes.

## Introduction

Pathogens often invade the hemocoel of a mosquito, but they are immediately confronted by two forces: hemolymph currents and immune responses^1,2^. The dorsal vessel is the primary circulatory organ that maintains hemolymph currents, and it extends the entire length of the body and is subdivided into an aorta in the thorax and a heart in the abdomen^3^. Contraction of the heart aspirates hemolymph into the lumen of the dorsal vessel via six pairs of abdominal ostia (heart valves) and one pair of thoraco-abdominal ostia. These contractions then propel the hemolymph to the anterior and posterior ends of the body^4–6^.

The primary drivers of immune responses in the hemocoel are the hemocytes^7^. In mosquitoes, three quarters of hemocytes circulate with the hemolymph whereas one quarter attaches to tissues and is sessile^8^. The distribution of sessile hemocytes is not homogenous. Many hemocytes aggregate in the areas surrounding the heart’s ostia, where they are called periostial hemocytes^9^. During an infection, periostial hemocytes sequester and kill pathogens that are about to be swept into the heart by the flow of hemolymph^9,10^. As this is happening, additional hemocytes aggregate at the periostial regions, which amplifies the pathogen killing response^9,11^. As a result, the most intense immune responses and the highest concentration of hemocytes coincide with the regions of the body that receive the most hemolymph flow^9,11^. Therefore, periostial hemocyte aggregation highlights the functional integration between the immune and circulatory systems^3,12^.

The periostial response to infection is not restricted to mosquitoes. Infection induces hemocyte aggregation on the heart of insects from all major branches of the class Insecta, which illustrates the evolutionary conservation of this immune process^13^. Despite its significance in insect immunity, the genetic factors that govern heart-associated immune responses remain largely unknown. Genes belonging to the Nimrod gene family–*Eater* and *Draper*–and the Thioester-containing protein gene family–*TEP1*, *TEP3* and *TEP4*—regulate periostial hemocyte aggregation in the mosquito, *Anopheles gambiae*^14,15^. Moreover, the IMD and JNK pathways—two of the major innate immune signaling pathways—also positively regulate periostial hemocyte aggregation^16^. The latter study also discovered that the mRNA abundance of two of the three transglutaminase genes in *A. gambiae*—*TGase1* and *TGase3*—is enriched in the periostial hemocytes of infected mosquitoes relative to the circulating hemocytes or the rest of the body^16^. Based on these data, we hypothesized that transglutaminases are also involved in heart-associated immune responses.

Transglutaminases catalyze the formation of isopeptide bonds between glutamine and lysine residues, and are encoded in the genomes of bacteria, plants and animals^17–19^. Transglutaminases in *Drosophila melanogaster* perform pleiotropic functions, including cuticular morphogenesis, hemolymph coagulation, pathogen entrapment, and peritrophic matrix formation^20,21^. Transglutaminases in other arthropods have similar functions, and of interest to the present study is that they coagulate wounds in horseshoe crabs^22,23^ and crayfish^24,25^, and are involved in immune defenses against viruses, bacteria and fungi in shrimps^26,27^, crabs^28^ and termites^29,30^.

The *A. gambiae* genome encodes three transglutaminase genes: *TGase1*, *TGase2* and *TGase3*^31^. The function of *TGase1* remains unknown. However, *TGase2* functions in the wound-induced antimalarial response^32,33^, and *TGase3* cross-links seminal secretions to form a gelatinous mating plug that is transferred to the female during mating and is essential for sperm storage in the spermatheca^31,34,35^. Here, we investigated whether transglutaminases are involved in heart-associated immune responses. We show that *TGase3*—but not *TGase1* or *TGase2*—negatively regulates periostial hemocyte aggregation during the early stages of infection, indicating that it plays a role in the functional integration between the mosquito immune and circulatory systems.

## Results

### Infection induces the expression of transglutaminase genes

In a separate study, we conducted RNAseq on periostial hemocytes, circulating hemocytes and abdomen of uninfected and infected mosquitoes at 4 h post-infection^16^. This 4 h timepoint was selected because the number of periostial hemocytes approximately doubles within the first hour of infection, and plateaus by 4 h after infection^9^. Mining that RNAseq data uncovered that an infection with GFP-*E. coli* or *S. aureus* more highly upregulates the expression of *TGase1* and *TGase3* in periostial hemocytes than in other tissues (Table 1).

**Table 1:** Regulation of transglutaminase gene expression in the periostial hemocytes, circulating hemocytes and abdomen at 4 h following GFP-*E. coli* or *S. aureus* infection^a^.

			*E. coli* versus Injured		*S. aureus* versus Injured	
Gene ID	Gene	Tissue	Log2 Fold Change	Adjusted P	Log2 Fold Change	Adjusted P
AGAP009100	*TGase1*	Heart and periostial hemocytes	3.20	3E-37	3.76	1E-51
AGAP009100	*TGase1*	Circulating hemocytes	1.38	7E-07	2.03	2E-15
AGAP009100	*TGase1*	Abdomen	1.39	1E-06	2.58	1E-23
AGAP009098	*TGase2*	Heart and periostial hemocytes	1.95	2E-08	2.40	1E-12
AGAP009098	*TGase2*	Circulating hemocytes	0.36	0.4869	0.99	0.0102
AGAP009098	*TGase2*	Abdomen	1.58	4E-05	1.70	4E-06
AGAP009099	*TGase3*	Heart and periostial hemocytes	3.98	2E-17	4.05	1E-18
AGAP009099	*TGase3*	Circulating hemocytes	1.41	0.0101	2.05	2E-05
AGAP009099	*TGase3*	Abdomen	1.12	0.1204	2.19	3E-05

^a^Data mined from SRA data PRJNA730047 in NCBI (https://www.ncbi.nlm.nih.gov).

**Figure 1:**
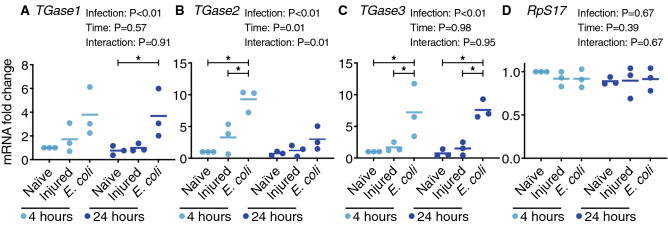
Transglutaminase genes are transcriptionally upregulated following infection. Graphs show the relative mRNA level of *TGase1* (**A**), *TGase2* (**B**), *TGase3* (**C**) and *RpS17* (**D**; negative control; *RpS17* data is shared with a concurrently conducted study^16^), in naïve (unmanipulated), injured and *E. coli-*infected mosquitoes at 4 or 24 h post-treatment. Each circle represents an independent biological trial, and the value is the average mRNA abundance relative to naïve mosquitoes at 4 h across 2–3 technical replicates within the trial. Data were analyzed by two-way ANOVA followed by Tukey’s post-hoc test. Infection *P* indicates whether infection has an effect irrespective of time, time *P* indicates whether time has an effect irrespective of infection, and interaction P indicates whether the effect of infection changes with time. The horizontal lines mark the means, and asterisks indicate post-hoc *P* < 0.05.

To explore this further, we examined by qPCR the expression of all three members of the transglutaminase family—*TGase1* (AGAP009100), *TGase2* (AGAP009098) and *TGase3* (AGAP009099)—in the whole body of female mosquitoes that were naïve, injured, or had been infected with tetracycline resistant, GFP-expressing *Escherichia coli* (GFP-*E. coli*) for 4 or 24 h (Fig. 1). For *TGase1*, mRNA abundance was similar between naïve and injured mosquitoes, but increased 3.8- and 4.8-fold after infection at 4 and 24 h, relative to naïve mosquitoes at 4 and 24 h, respectively. For *TGase2*, injury induced a 3.3-fold increase in expression at 4 h but only marginally changed expression at 24 h, and infection increased the mRNA abundance by 9.3- and 4.0-fold at 4 and 24 h, respectively, relative to naïve mosquitoes at the same timepoint. For *TGase3*, mRNA abundance doubled following injury but increased 7.2- and 10.4-fold after infection for 4 and 24 h, relative to naïve mosquitoes at 4 and 24 h, respectively. As expected, the mRNA abundance of the control gene, *RpS17*, remained unchanged regardless of treatment and time. Overall, these data show that injury upregulates the expression of *TGase2*, while infection upregulates the expression of *TGase1*, *TGase2* and *TGase3*. Together, with the RNAseq data on periostial hemocytes, circulating hemocytes and abdomen (Table 1), these qPCR data led us to explore whether the three transglutaminases encoded in the *A. gambiae* genome are involved in heart-associated immune responses.

### *TGase3* negatively regulates infection-induced periostial hemocyte aggregation during the early stages of infection

To determine whether transglutaminases play a role in periostial hemocyte aggregation, we individually knocked down the expression of *TGase1*, *TGase2* or *TGase3* by RNA interference (RNAi) and evaluated the number of periostial hemocytes before infection and at 4 and 24 h after GFP-*E. coli* infection. The RNAi-based knockdown efficiency for *TGase1*, *TGase2* and *TGase3* was 57%, 79% and 41%, respectively, compared to the control ds*bla(Ap*^*R*^*)*-injected mosquitoes (Supplementary Fig. S1).

**Figure 2:**
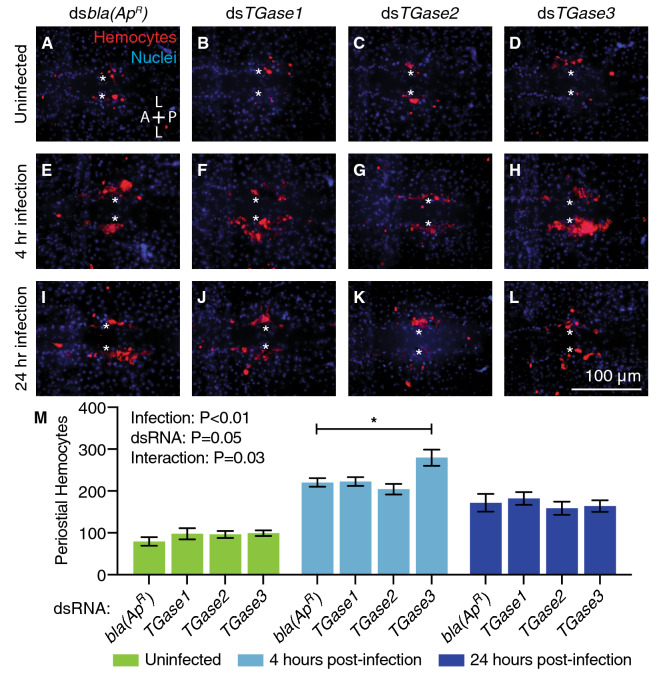
RNAi-based knockdown of *TGase3* increases the number of periostial hemocytes during the early stages of GFP-*E. coli* infection. (**A**–**L**) Each fluorescence image shows the tergum of a single abdominal segment with periostial hemocytes (CM-DiI; red) surrounding the ostia (asterisks) in uninfected mosquitoes (**A**–**D**), and mosquitoes at 4 (**E**–**H**) or 24 h (**I**–**L**) post-infection. Prior to treatment, mosquitoes had been injected with ds*bla(Ap*^*R*^*)* (**A**, **E**, **I**), ds*TGase1* (**B**, **F**, **J**), ds*TGase2* (**C**, **G**, **K**) or ds*TGase3* (**D**, **H**, **L**). Nuclei were stained blue with Hoechst 33342. A, anterior; P, posterior; L, lateral. (**M**) Graph shows the average number of periostial hemocytes in ds*bla(Ap*^*R*^*)-*, ds*TGase1-*, ds*TGase2-* and ds*TGase3-*injected mosquitoes that were uninfected or had been infected with GFP-*E. coli* for 4 or 24 h. Data were analyzed by two-way ANOVA followed by Dunnett’s post-hoc test, using ds*bla(Ap*^*R*^*)* mosquitoes as the reference. Infection *P* indicates whether infection has an effect irrespective of dsRNA treatment, dsRNA *P* indicates whether dsRNA treatment has an effect irrespective of infection, and interaction *P* indicates whether the effect of infection changes with dsRNA treatment. Column heights mark the means, whiskers show the standard error of the mean (S.E.M.), and the asterisk indicates post-hoc *P* < 0.05.

In ds*bla(Ap*^*R*^*)*-injected mosquitoes, infection for 4 h increased the number of periostial hemocytes 2.8-fold relative to their naïve counterparts, and this number remained elevated at 24 h after infection (Fig. 2). This reiterates that infection induces periostial hemocyte aggregation^8,9,11^. When transglutaminases were knocked down, the number of periostial hemocytes in uninfected mosquitoes was similar between the experimental and control groups, indicating that transglutaminases do not regulate the basal abundance of periostial hemocytes. When infected for 4 or 24 h, the number of periostial hemocytes in ds*TGase1* and ds*TGase2* mosquitoes increased in a manner that was similar to the increase seen for ds*bla(Ap*^*R*^*)* mosquitoes, indicating that knocking down these two genes does not affect periostial hemocyte aggregation. However, the number of periostial hemocytes at 4 h post-infection in ds*TGase3* mosquitoes was 27% higher than in ds*bla(Ap*^*R*^*)* mosquitoes, although this effect disappeared by 24 h.

When the spatial distribution of hemocytes along the six periostial regions was assayed, there was no interaction between the type of dsRNA treatment and the proportional distribution of periostial hemocytes along the length of the abdomen (Supplementary Fig. S2). Specifically, there were always fewer hemocytes in the periostial regions of segments 2 and 3, and most hemocytes tended to be in the periostial regions of segments 4–6. This spatial distribution correlates with more periostial hemocytes being present in the regions of the heart that receive the most hemolymph flow^11^. Overall, these data show that *TGase3* negatively regulates infection-induced periostial hemocyte aggregation during the early stages of infection but does not alter the proportional distribution of hemocytes amongst the different periostial regions.

### There is a trend for *TGase3* to positively regulate non-periostial, sessile hemocyte abundance in uninfected mosquitoes

**Figure 3:**
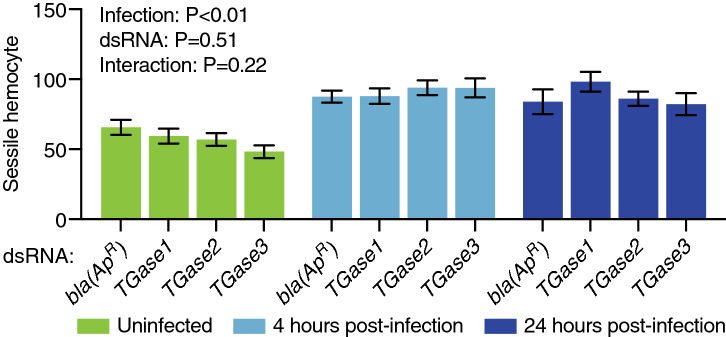
RNAi-based knockdown of transglutaminase genes has a limited effect on the aggregation of sessile hemocytes outside of the periostial regions. Graph shows the average number of non-periostial sessile hemocytes on the integument of dorsal abdominal segments 4 and 5 in ds*bla(Ap*^*R*^*)-*, ds*TGase1-*, ds*TGase2-* and ds*TGase3-*injected mosquitoes that were uninfected or had been infected with GFP-*E. coli* for 4 or 24 h. Data were analyzed by two-way ANOVA, using ds*bla(Ap*^*R*^*)* mosquitoes as the reference. Column heights mark the means, and whiskers show the S.E.M.

We next counted the number of non-periostial sessile hemocytes in the dorsal tergum of abdominal segments 4 and 5 in the same mosquitoes that were examined for periostial hemocytes. The rationale for collecting this measurement was to determine whether transglutaminases affect the general abundance of sessile hemocytes, and more specifically, whether the effect seen for *TGase3* at 4 h after infection was a general phenomenon that affects all sessile hemocytes, or whether it is specific to the periostial regions. In uninfected mosquitoes, and relative to ds*bla(Ap*^*R*^*)* injection, knockdown of *TGase3* reduced the number of non-periostial sessile hemocytes by 26% (*P* = 0.07), whereas knockdown of *TGase1* or *TGase2* had a minimal effect on the number of non-periostial hemocytes (Fig. 3). Infection induced a modest increase in the number of non-periostial sessile hemocytes, but this increase was unaffected by dsRNA treatment (Fig. 3). The magnitude of the infection-induced increase in non-periostial sessile hemocytes was small relative to the increase seen in the periostial regions.

### Transglutaminases do not modulate the phagocytosis of bacteria on the surface of the heart

**Figure 4:**
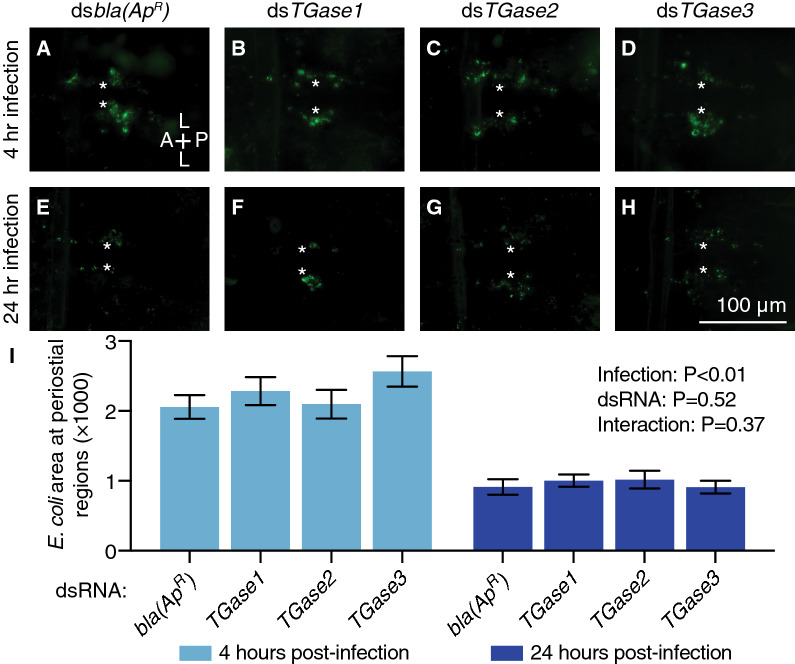
RNAi-based knockdown of transglutaminase genes does not alter the accumulation of GFP-*E. coli* in the periostial regions. (**A–H**) Fluorescence images show a single abdominal segment with phagocytosed bacteria (green) surrounding the ostia (asterisks) in mosquitoes that had been infected with GFP-*E. coli* for 4 (**A**–**D**) or 24 h (**E**–**H**). Prior to treatment, mosquitoes had been injected with ds*bla(Ap*^*R*^*)* (**A**, **E**), ds*TGase1* (**B**, **F**), ds*TGase2* (**C**, **G**) or ds*TGase3* (**D**, **H**). A, anterior; P, posterior; L, lateral. (**I**) Graph shows the average area of GFP-*E. coli* in ds*bla(Ap*^*R*^*)-*, ds*TGase1-*, ds*TGase2-* and ds*TGase3-*injected mosquitoes that had been infected with GFP-*E. coli* for 4 or 24 h. Data were analyzed by two-way ANOVA, using ds*bla(Ap*^*R*^*)* mosquitoes as the reference. Column heights mark the means, and whiskers show the S.E.M.

Hemocytes phagocytose and destroy pathogens that circulate with the hemolymph, and this phagocytic activity is strongly manifested at the periostial regions^1,9–11^. Given that *TGase3* knockdown increases the infection-induced aggregation of periostial hemocytes during the early stages of infection, we next sought to assay whether knocking down transglutaminases affects the accumulation of phagocytosed GFP-*E. coli* at the periostial regions. To our surprise, the GFP*-E. coli* pixel area in the periostial regions was similar between the ds*bla(Ap*^*R*^*)*-injected mosquitoes and the ds*TGase1*-, ds*TGase2*- and ds*TGase3*-injected mosquitoes at both 4 and 24 h post-infection (Fig. 4). For all dsRNA groups, the presence of fluorescent bacteria at the periostial regions significantly dropped between 4 and 24 h following infection, which shows that phagocytosed bacteria are destroyed and that the immune response at the periostial regions is effective. Overall, these data show that transglutaminases do not modulate the hemocyte-mediated phagocytosis of bacteria at the periostial regions.

### *TGase3* negatively regulates melanin accumulation on the surface of the heart during the later stages of infection

**Figure 5:**
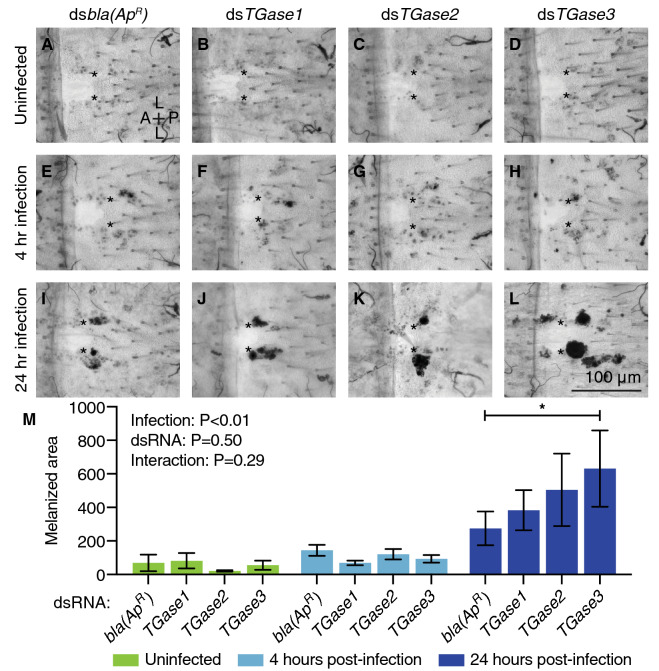
RNAi-based knockdown of *TGase3* increases melanin accumulation at the periostial regions. (**A–L**) Brightfield images show a single abdominal segment with melanin deposits (black) surrounding the ostia (asterisks) in uninfected mosquitoes (**A**–**D**), and mosquitoes that had been infected with GFP-*E. coli* for 4 (**E**–**H**) or 24 h (**I**–**L**). Prior to treatment, mosquitoes had been injected with ds*bla(Ap*^*R*^*)* (**A**, **E**, **I**), ds*TGase1* (**B**, **F**, **J**), ds*TGase2* (**C**, **G**, **K**) or ds*TGase3* (**D**, **H**, **L**). A, anterior; P, posterior; L, lateral. (**M**) Graph shows the average area of melanin deposits in ds*bla(Ap*^*R*^*)-*, ds*TGase1-*, ds*TGase2-* and ds*TGase3-*injected mosquitoes that were not infected or had been infected with GFP-*E. coli* for 4 or 24 h. Data were analyzed by two-way ANOVA followed by Dunnett’s post-hoc test, using ds*bla(Ap*^*R*^*)* mosquitoes as the reference. Column heights mark the means, whiskers show the S.E.M., and the asterisk indicates post-hoc *P* < 0.05.

Hemocytes produce enzymes that catalyze the melanization cascade that encases and kills pathogens^36–40^. Melanization is a humoral response that occurs in circulation, but this response leads to the eventual phagocytosis of dark, melanized pathogens by periostial hemocytes and the capture of smaller, melanized debris by pericardial cells^9,11,15,41,42^. Given that *TGase3* knockdown increases the number of periostial hemocytes at 4 h after infection but does not affect the phagocytosis of live bacteria, we tested whether transglutaminases are involved in the accumulation of melanin on the surface of the heart. To achieve this, we measured the cumulative area of dark melanin deposits within the periostial regions of the same mosquitoes examined for GFP-*E. coli* accumulation. Melanin was undetectable in uninfected mosquitoes and minimal in mosquitoes that had been infected with GFP-*E. coli* for 4 h. However, at 24 h post-infection, knockdown of *TGase3* increased the melanized area within the periostial regions when compared to ds*bla(Ap*^*R*^*)*-injected mosquitoes (Fig. 5). Knockdown of *TGase1* and *TGase2* also slightly increased the melanized area, but this difference was smaller and not statistically significant. Overall, these data show that *TGase3* negatively regulates the accumulation of melanized bacteria at the periostial regions.

## Discussion

**Figure 6:**
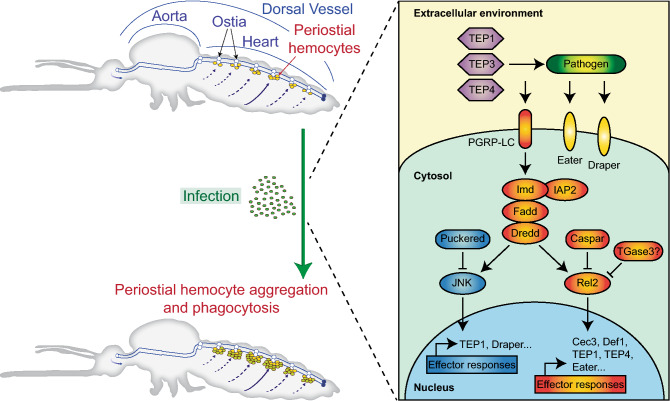
Proposed molecular regulation of infection-induced periostial hemocyte aggregation. Infection induces the aggregation of hemocytes in the periostial regions of the heart. This process is positively regulated by the IMD and JNK pathways, thioester containing proteins (TEPs) and nimrod family proteins (Eater and Draper), and is negatively regulated by caspar, puckered and TGase3.

Here we show that *TGase3* plays a negative role in periostial hemocyte aggregation during the early stages of infection. In contrast, we also detected a trend that *TGase3* positively regulates sessile hemocyte attachment to the abdominal integument in uninfected mosquitoes. Based on these and other data^16,43,44^, we hypothesize that *TGase3* negatively regulates periostial hemocyte aggregation by inhibiting the immune deficiency (IMD) pathway and altering hemocyte adhesion (Fig. 6).

In *D. melanogaster*, infections activate the IMD pathway, which leads to the cleavage of the NF-κB transcription factor, Relish. The N-terminal region of Relish translocates into the nucleus, binds to DNA, and induces the expression of antimicrobial peptides and the activation of other immune responses that kill pathogens^45^. Interestingly, *Drosophila* transglutaminase negatively regulates the IMD pathway by crosslinking the N-terminal region of Relish and forming a polymer that prevents its translocation into the nucleus^43,44^. This transglutaminase also incorporates natural primary amines into the DNA binding site of Relish, thereby providing a second layer of IMD pathway inhibition^44^. Here, we did not test the direct relationship between *A. gambiae TGase3* and the IMD pathway. However, we show that *TGase3* negatively regulates periostial hemocyte aggregation during the early stages of infection and melanin accumulation in the periostial regions during the later stages of infection. Moreover, in an earlier study we demonstrated that the IMD pathway positively regulates periostial hemocyte aggregation^16^. Specifically, silencing the *A. gambiae* ortholog of *D. melanogaster Relish*, *rel2*, reduces periostial hemocyte aggregation whereas silencing the negative regulator of the IMD pathway, *caspar*, increases the periostial immune response. Therefore, given that (i) *TGase3* is a negative regulator of periostial hemocyte aggregation, (ii) the IMD pathway is a positive regulator of periostial hemocyte aggregation, and (iii) transglutaminase inhibits the transcription factor that positively regulates the IMD pathway, we hypothesize that mosquito *TGase3* negatively regulates periostial hemocyte aggregation by inhibiting the IMD pathway.

Silencing *TGase3* also slightly reduced the number of sessile hemocytes in uninfected mosquitoes. A plausible explanation is that silencing *TGase3* causes sessile hemocytes to detach from the abdominal wall, trachea and other tissues, thus forcing them into circulation. This would increase the number of circulating hemocytes, thereby making more of these cells available to aggregate in the periostial regions of infected mosquitoes. Whether *TGase3* regulates hemocyte attachment in mosquitoes is unknown, but two human transglutaminase homologs—Factor XIII and tissue transglutaminase 2 (hTGase2)—are involved in cell-substrate binding^46–50^. Moreover, in a freshwater crayfish, transglutaminase is expressed in hematopoietic cells and functions to maintain hemocytes within the hematopoietic organ^51^. In another crustacean, the white shrimp, reducing transglutaminase expression increases the circulating hemocyte population, possibly because of the detachment of sessile hemocytes^27^. Overall, this suggests that reduced levels of transglutaminase are associated with the release of sessile hemocytes into circulation. This may explain why mosquitoes with silenced *TGase3* tended to have fewer non-periostial, sessile hemocytes than mosquitoes with a natural amount of TGase3.

In this study, we found that *TGase1* and *TGase2* are not involved in periostial hemocyte aggregation. This was expected for *TGase2* because it is involved in wound-induced immunity^32^, and injury does not induce periostial hemocyte aggregation^8,9,11^. However, we had hypothesized that *TGase1* would be involved in periostial hemocyte aggregation. This was because infection upregulates the expression of both *TGase1* and *TGase3* more highly in the heart with periostial hemocytes than in other tissues^16^, but perhaps the lack of involvement of *TGase1* in heart-associated immune responses is congruent with a previous study that found that *TGase1* does not participate in the immune response against malaria parasites, at least in the gut^32^. However, it remains possible that *TGase1* has an alternate function in periostial hemocytes or that *TGase1* is involved in heart physiology; *Drosophila* transglutaminase is highly expressed in the ostia of the embryo and adults^52,53^.

Here we show that *TGase3* is a negative regulator of periostial hemocyte aggregation. Prior to this study, *TGase3* was only known to be essential for the formation of the anopheline-specific mating plug^31^. A previous study suggested that *TGase3* is expressed in the male accessory glands of *A. gambiae*, but that it is not expressed in female mosquitoes^31^. However, we previously detected *TGase3* expression in the heart, periostial hemocytes and whole body of female mosquitoes by RNAseq^16^, and did so again here by quantitative RT-PCR and conventional RT-PCR. Because *TGase3* is involved in periostial hemocyte aggregation it is possible that it may be involved in the immune response against malaria sporozoites. Sporozoites inside midgut oocysts are released into the hemocoel and passively migrate with the flow of hemolymph until they reach and invade the salivary glands^10,54^. Some of these sporozoites are captured and destroyed by periostial hemocytes^9,10^, and therefore, the role that *TGase3* plays against the sporogonic stages of *Plasmodium* parasites should be investigated. Altogether, this suggests that the three transglutaminases in *A. gambiae* have different functions. Based on our limited knowledge of TGase1-3, we conclude that (i) *TGase1* plays a lesser role or no role in immunity, (ii) *TGase2* is involved in wound-related immunity, and (iii) *TGase3* is involved in both mating biology and periostial hemocyte aggregation. Alternatively, a possible explanation for the lack of a phenotype in *TGase1* or *TGase2* RNAi mosquitoes is that the residual amount of protein that remained after partial RNAi-based silencing prevented us from observing a phenotype shift, or that there is a redundant mechanism involving *TGase3*. This would not be entirely surprising because our treatments were conducted four days after the onset of RNAi, and *Drosophila* transglutaminase can still be detected 12 days after the initiation of RNAi^43^.

Transglutaminases participate in many physiological processes. Here we show that *TGase3* is involved in heart-associated immune responses. Based on what is already known about arthropod transglutaminases, we hypothesize that this occurs via two complementary mechanisms: (i) modification of the infection-induced activation of the IMD pathway and (ii) modulation of hemocyte adhesion. In conclusion, this study shows that a factor that is not classically associated with immunity or circulatory physiology is involved in the functional integration of the immune and circulatory systems of mosquitoes.

## Methods

### Mosquitoes, bacteria, and infection

*Anopheles gambiae*, Giles sensu stricto (G3 strain; Diptera: Culicidae), were reared and maintained in an environmental chamber at 27 °C and 75% relative humidity, with a 12 h:12 h light:dark photoperiod^4^. Adults were fed 10% sucrose ad libitum, and only females were used for experimentation. GFP-*E. coli* (modified DH5α) were grown overnight in Luria–Bertani’s (LB) rich nutrient medium in a 37 °C shaking incubator (New Brunswick Scientific, Edison, NJ, USA). On average, 17,388 GFP-*E. coli* were injected into the thoracic anepisternal cleft using a Nanoject III Programmable Nanoliter Injector (Drummond Scientific Company, Broomall, PA, USA).

### Treatments, RNA extraction and cDNA synthesis

Six-day-old mosquitoes were cold-anesthetized and separated into three groups: (1) naïve (unmanipulated), (2) injured by injecting 69 nL of sterile LB medium, and (3) infected by injecting 69 nL of GFP-*E. coli*. RNA was extracted and isolated from a pool of 10 whole-body mosquitoes at 4 and 24 h after treatment using TRIzol Reagent (Invitrogen, Carlsbad, CA, USA), and purified using the RNeasy Mini Kit (Qiagen, Valencia, CA, USA). The concentration of RNA was quantified using a BioPhotometer Plus spectrophotometer (Eppendorf AG, Hamburg, Germany), and up to 5 μg of RNA was treated with RQ1 RNase-free DNase (Promega, Madison, WI, USA). The extracted RNA was then used for cDNA synthesis using Oligo(dT)_20_ primers and the SuperScript III First-Strand Synthesis System for RT-PCR (Invitrogen).

### Gene expression and real-time quantitative PCR (qPCR)

qPCR was conducted using cDNA as template, gene-specific primers (Supplementary Table S1), and Power SYBR Green PCR Master Mix (Applied Biosystems, Foster City, CA) on a Bio Rad CFX Connect Real-Time Detection System (Hercules, CA, USA). Relative quantification of mRNA levels was conducted using the 2^-ΔΔC^_T_ method, with the housekeeping gene *RpS7* as the reference gene and *RpS17* as a control^55,56^. The mRNA fold change was relative to naïve mosquitoes at 4 h. Three independent trials were conducted, each with 2–3 technical replicates. The absence of genomic DNA in the cDNA preparations was confirmed by (i) conducting a melting curve analysis at the end of each qPCR run to show that there is only one peak, and (ii) testing each cDNA with primers that span an intron to show that only one amplicon that is of the predicted mRNA size amplified. Gene expression analyses for this study and for another recent study were conducted concurrently and using the same cDNAs, and hence, the *RpS17* data are the same for both studies^16^.

### Double-stranded RNA (dsRNA) synthesis

Double-stranded RNA was synthesized for three mosquito genes—*TGase1*, *TGase2* and *TGase3*—using methods we have described^14,15,57^. Specifically, a fragment of each gene was amplified using *A. gambiae* cDNA as the template and gene-specific primers with T7 promoter tags (Supplementary Table S1). The amplicons were separated by agarose gel electrophoresis, excised, and purified using Qiagen’s QIAquick Gel Extraction kit. Each amplicon was then used as the template for a second PCR reaction using the same primers. The product was purified using the QIAquick PCR Purification Kit (Qiagen), and the concentration was quantified spectrophotometrically. Up to 1 μg of the second PCR product was used as the template for in vitro dsRNA synthesis using the MEGAscript T7 Kit (Applied Biosystems). The resultant dsRNA was precipitated with ethanol and re-suspended in phosphate-buffered saline (PBS). The concentration of dsRNA was quantified spectrophotometrically and the integrity of dsRNA was verified by agarose gel electrophoresis. The same procedure was used to synthesize dsRNA for a non-mosquito gene—*bla(Ap*^*R*^*)* (beta-lactamase)—except that the template was DNA extracted from *E. coli* BL21(DE3) containing the pET-46 plasmid (EMD Chemicals, Gibbstown, NJ).

### RNA interference

Two-day-old female mosquitoes were injected with 300 ng of dsRNA. Four days later, mosquitoes were divided into two groups: (1) uninfected and (2) infected with GFP-*E. coli*. An injury group (e.g., sham injection) was not assayed because injury does not induce periostial hemocyte aggregation^8,9,11^. To assess RNAi efficiency, RNA was purified from whole bodies at 4 days after dsRNA injection, cDNA was synthesized, and qPCR was conducted with gene-specific primers (Supplementary Table S1). The mRNA fold change (silencing efficiency) was relative to ds*bla(Ap*^*R*^*)* mosquitoes. For qPCR, three to four independent trials were conducted with two technical replicates each.

### Hemocyte staining and mosquito dissections

Hemocytes were stained in vivo using Vybrant CM-DiI Cell-Labeling Solution (Invitrogen) as we have previously described^9^. Briefly, live mosquitoes were injected with ~ 0.4 μL of a solution consisting of 67 μM CM-DiI and 1.08 mM Hoechst 33342 (nuclear stain; Invitrogen) in PBS. Mosquitoes were incubated in the environmental chamber for 20 min, and then fixed by injecting 16% paraformaldehyde into the hemocoel. Ten minutes later, a razor blade was used to separate the abdomen from the head and thorax, and to bisect the abdomen along a coronal plane such that the dorsal and ventral sides were separated. The dorsal abdomens were then immersed in PBS containing 0.1% Triton X-100 and the internal organs were removed. The dorsal abdomens—containing the heart, periostial hemocytes and pericardial cells—were rinsed briefly in PBS and mounted between a glass slide and a coverslip using Aqua-Poly/Mount (Polysciences; Warrington, PA, USA).

### Microscopy and image acquisition

Each dorsal abdomen was imaged using a Nikon Eclipse Ni-E compound microscope connected to a Nikon Digital Sight DS-Qi1 monochrome digital camera and Nikon’s Advanced Research NIS Elements software (Nikon, Tokyo, Japan). Z-stacks of bright-field, red fluorescence (hemocytes), green fluorescence (GFP-*E. coli*) and blue fluorescence (nuclei) channels were acquired using a linear encoded Z-motor. For image presentation and pixel measurements, a specific channel (or channels) was (were) selected and all images within a stack were combined into a two-dimensional, focused image using the Extended Depth of Focus (EDF) function in NIS Elements.

### Hemocyte counting

Hemocytes were counted manually by examining all images within a Z-stack. A cell was counted as a hemocyte if it measured 9–18 μm in diameter and was labeled with both CM-DiI and Hoechst 33342^11^. A cell was counted as a periostial hemocyte if it was adjacent to an ostium, and a cell was counted as a non-periostial, sessile hemocyte if it was attached to the dorsal abdominal wall in an area that was outside of the periostial regions^8,9,15^. Periostial hemocytes were counted within abdominal segments 2–7 whereas non-periostial sessile hemocytes were only counted on the dorsal abdominal wall of segments 4 and 5. Hemocytes were not counted on the aorta, the excurrent openings of the heart or the thoraco-abdominal ostia because few hemocytes are present there, and infection does not induce the aggregation of hemocytes at those locations^5,6^. Each treatment group contained between 22 and 37 mosquitoes, which were assayed across 3–4 independent biological trials. Periostial and non-periostial sessile hemocytes were counted in the same specimens.

### Quantification of GFP-*E. coli* at the periostial regions

GFP-*E. coli* in the periostial regions was quantified by measuring the area of pixels with intensities above a threshold. Images were first examined to determine a pixel intensity threshold that distinguished GFP emitted by *E. coli* (pixel intensities above the threshold) from background fluorescence intensity (pixel intensities below the threshold). Then, each of the periostial regions was delineated using the region of interest (ROI) tool in NIS Elements, and the sum binary area of pixels above the threshold was measured for each periostial region ROI. Each treatment group contained between 22 and 38 mosquitoes, which were assayed across 3–4 independent trials.

### Quantification of melanin at the periostial regions

Dark melanin deposits in the periostial regions were quantified by measuring the area of pixels with intensities below a threshold^11^. Images were first examined to determine a threshold of pixel intensity that distinguished melanized areas (pixel intensities below the threshold) from non-melanized areas (pixel intensities above the threshold). Then, each periostial region was delineated using the ROI tool, and the sum binary area of pixels below the threshold was measured for each periostial region ROI. Each treatment group contained between 22 and 38 mosquitoes, which were assayed across 3–4 independent trials. Phagocytosis (GFP-*E. coli*) and melanization were quantified in the same mosquitoes (and ROIs).

### Statistical analysis

Data on gene expression or the aggregation of hemocytes, pathogens or melanin were analyzed by two-way ANOVA, followed by Tukey’s or Dunn’s multiple comparison test. For aggregation, the ds*bla(Ap*^*R*^*)*-injected mosquitoes were used as the reference group. A two-way ANOVA yields three distinct *P*-values, which in the aggregation experiments examine: (1) whether dsRNA treatment affects the outcome, regardless of infection status; (2) whether infection status affects the outcome, regardless of dsRNA treatment; (3) whether the infection status has effects that depend on the dsRNA treatment, and vice versa. All data analysis was done in GraphPad Prism version 8.4.3, and differences were deemed significant at *P* < 0.05.

## Supplementary Information

Supplementary Information 1.

Supplementary Information 2.

## Data Availability

The datasets generated and analyzed during the current study are available in an accompanying supplementary file.
