# Supplementary material for: Transglutaminase 3 negatively regulates immune responses on the heart of the mosquito, Anopheles gambiae
Source: Sci Rep. 2022 Apr 25;12:6715. doi: 10.1038/s41598-022-10766-z (PMC9038791; doi:10.1038/s41598-022-10766-z)
Supplement: Supplementary file 1 — Supplementary Information 1. [file 41598_2022_10766_MOESM1_ESM.pdf]

Transglutaminase 3 negatively regulates immune responses on the heart of the mosquito, *Anopheles gambiae*

Yan Yan, Abinaya Ramakrishnan, Tania Y. Estévez-Lao and Julián F. Hillyer *

Department of Biological Sciences, Vanderbilt University, Nashville, TN, USA

julian.hillyer@vanderbilt.edu

Scientific Reports, 2022

File contents:

Table S1. Gene names, gene IDs, and primers used in this study.

Figure S1. RNAi-based knockdown efficiency of *TGase1*, *TGase2* and *TGase3*.

Figure S2. RNAi-based knockdown of transglutaminase genes does not alter the relative spatial distribution of periostial hemocytes.

Table S1. Gene names, gene IDs, and primers used in this study.

Gene	VectorBase ID[a]	Application	Sequence (forward and reverse)[b]	Amplicon (bp)[c]	
				Transcript	Genomic
RpS7	AGAP010592	qPCR	GACGGATCCCAGCTGATAAA	132	281
		qPCR	GTTCTCTGGGAATTCGAACG		
RpS17	AGAP004887	qPCR	GACGAAACCACTGCGTAACA	153	264
		qPCR	TGCTCCAGTGCTGAAACATC		
TGase1	AGAP009100	qPCR	CTGCACAAGGGACTGTTCCA	191	259
		qPCR	AACGCCAAAAAGCCATCCAC		
TGase2	AGAP009098	qPCR	CGGTGGACGCTGACTATCAA	225	297
		qPCR	GTACTGGCCGAGCTTCCATT		
TGase3	AGAP009099	qPCR	TACAGCAGCCAGCGGTTTAG	236	236
		qPCR	ATATCGCGCCCAGTGTAGTC		
bla(Ap^{R})	(Bacterial gene)	RNAi	TAATACGACTCACTATAGGGCCGAGCGCAGAAGTGGT	214	214
		RNAi	TAATACGACTCACTATAGGGAACCGGAGCTGAATGAA		
TGase1	AGAP009100	RNAi	TAATACGACTCACTATAGGGCATTCCGGTTAATCAGT	361	433
		RNAi	TAATACGACTCACTATAGGGCGTAGTCGATTGTAAGA		
TGase2	AGAP009098	RNAi	TAATACGACTCACTATAGGGTCAGAGCTGTCTAACAAA	490	490
		RNAi	TAATACGACTCACTATAGGCGTACCGCTCAACTCC		
TGase3	AGAP009099	RNAi	TAATACGACTCACTATAGGGAAAACCTTCCACACGTC	501	501
		RNAi	TAATACGACTCACTATAGGGTTGAACAGCACAAACAA		

[a] Vectorbase IDs were obtained from the AgamP4 assembly in www.vectorbase.org (exception: *bla(Ap^{R})*).
[b] Underlined sequences are specific to the T7 RNA polymerase promoter sites needed for dsRNA synthesis.
[c] Amplicon sizes are based on the sequences in Vectorbase. For dsRNA primers, amplicon lengths include the T7 promoter sequence tags.

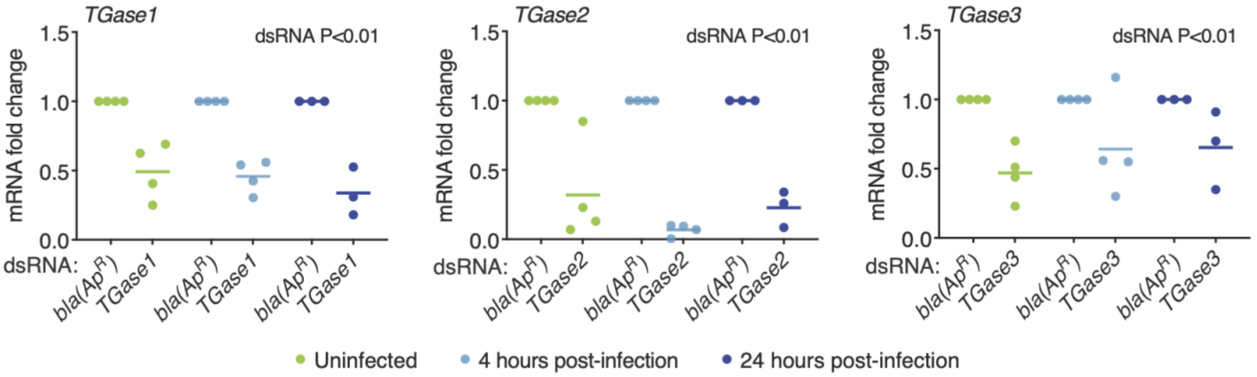

Supplementary Figure S1. RNAi-based knockdown efficiency of *TGase1*, *TGase2* and *TGase3*. Graphs show the relative mRNA abundance in mosquitoes treated with ds*bla(Ap^R)*-, ds*TGase1*-, ds*TGase2*- and ds*TGase3* that were uninfected or had been infected with GFP-*E. coli* for 4 or 24 hr. Each circle is an independent biological trial, and the value is the average mRNA abundance relative to the ds*bla(Ap^R)* group across 2-3 technical replicates within that trial. The horizontal lines mark the means. Data were analyzed by two-way ANOVA, and the P-value indicating the effect of dsRNA treatment.

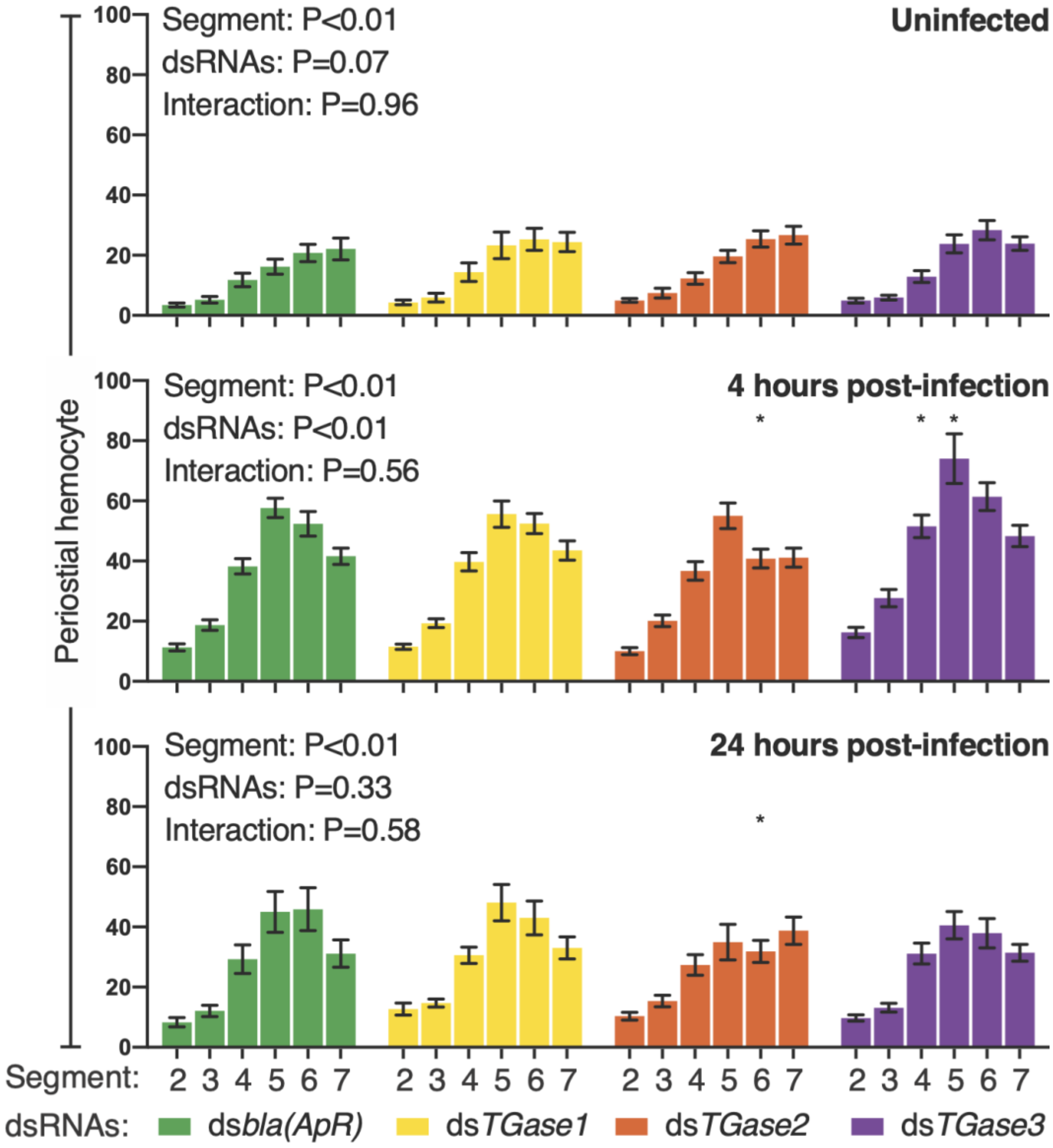

Supplementary Figure S2. RNAi-based knockdown of transglutaminase genes does not alter the relative spatial distribution of periostial hemocytes. Graph shows the average number of periostial hemocytes in each periostial region of abdominal segments 2-7 in ds*bla(Ap^R)*-, ds*TGase1*-, ds*TGase2*- and ds*TGase3*-injected mosquitoes that were uninfected or had been infected with GFP-*E. coli* for 4 or 24 hr. Data were analyzed by two-way ANOVA followed by Dunnett's post-hoc test, using ds*bla(Ap^R)* mosquitoes as the reference. Column heights mark the means and whiskers show the S.E.M. Asterisks indicate post-hoc P < 0.05, using ds*bla(Ap^R)* mosquitoes of the same segment as the reference.
